# Feeling Uncertainty during the Lockdown That Commenced in March 2020 in Greece

**DOI:** 10.3390/ijerph18105105

**Published:** 2021-05-12

**Authors:** Dimitris Zavras

**Affiliations:** Department of Public Health Policy, School of Public Health, University of West Attica, 11521 Athens, Greece; dzavras@uniwa.gr

**Keywords:** coronavirus disease 2019 pandemic, lockdown, uncertainty, age, income loss, job loss, social class, health literacy, concern for health

## Abstract

The coronavirus disease 2019 (COVID-19) pandemic has resulted in significant uncertainty for the global population. However, since not all population groups experience the impacts of the pandemic in the same way, the objective of this study was to identify the individual characteristics associated with the feeling of uncertainty during the lockdown that commenced in March 2020 in Greece. The study used data from the “Public Opinion in the European Union (EU) in Time of Coronavirus Crisis” survey. The sample consisted of 1050 individuals aged between 16 and 54 years. According to the analysis, which was based on a logistic regression model, the emotional status of older individuals, those who experienced income and job losses since the beginning of the pandemic, and middle-class and high-class individuals, is more likely to be described as a feeling of uncertainty. In addition, the emotional status of individuals with less concern for their own health and that of family and friends is less likely to be described as a feeling of uncertainty. Although the results related to age, income, and job losses, as regards concern for health, agree with the international literature, the limited health literacy of lower-class individuals may explain the reduced likelihood of their experiencing feelings of uncertainty. The results confirm the international literature describing several aspects of uncertainty due to the COVID-19 crisis.

## 1. Introduction

Because public health emergencies adversely affect the health, safety, and well-being of individuals and communities, they may provoke a range of emotional reactions [1]. Thus, it is not surprising that during the coronavirus disease 2019 (COVID-19) pandemic, a substantial proportion of society has felt uncertainty [2].

Uncertainty exists when details of situations are ambiguous, complex, unpredictable, or probabilistic, when information is unavailable or inconsistent, and when people feel insecure about their own state of knowledge or the state of knowledge in general [3].

Uncertainty in events is considered to be a consequence of a gap between knowledge of the actual occurrence of events in the real world and the availability of knowledge regarding these events [4], that is, uncertainty refers to a state characterized by a lack of information about whether, where, when, how, or why an event has occurred or will occur [5].

Since it is impossible to assign probabilities to the likelihood of future events, it is difficult to judge the future impact of uncertainty due to unpredictable or uncontrollable external events such as economic fluctuations [6] and pandemics [7].

Unpredictable events are complex and one of the main sources of uncertainty [8]; other sources of uncertainty include situations that are unfamiliar or not easily resolved and situations that are novel or insoluble. The period of anticipation prior to the confrontation with a potentially harmful event and the notion that negative events may occur without a definitive means of predicting them can also cause uncertainty [9].

During the period of the COVID-19 pandemic, most of these sources have contributed to the high degree of global uncertainty in the context of the pandemic’s physical, psychological, financial, and social impacts [10]. Uncertainty is therefore related not only to the seriousness of the threats to people’s physical health and lives, the lack of early knowledge about quarantine duration, the real risk of exposure, and the unpredictability of symptomatology, but also to the impacts on personal, economic, and societal levels [11]. Thus, uncertainty arises from various aspects of the COVID-19 crisis.

That is, in parallel with the uncertainties related to infectiousness, viral lethality [12], mutations [13], and prevention and treatment [14], the COVID-19 pandemic has created a pervasive atmosphere of general uncertainty, concerning both personal finances and the overall state of the economy and finance [15,16], as well as uncertainty concerning the costs and benefits of lockdown-lifting strategies [17].

Indeed, due to the pandemic, the global community has been facing not only a public health crisis, but also an economic crisis [18]. According to Kose and Sugawara, the current recession is the first, since 1870, to be driven solely by a pandemic [19].

Economic recessions cause individuals to have feelings of uncertainty that are associated with their finances, reduced income, or fear of losing their jobs [20], which have also been experienced during the current pandemic [21,22,23]; however, even though a person may not experience income or job losses, feelings of uncertainty may arouse as a result of working under pressure or being exposed to so many deaths [24].

In addition, feelings of uncertainty may be triggered by the risk of contracting the COVID-19 virus, which causes a potentially life-threatening infection [25,26].

Moreover, the stringent measures implemented to control the spread of the virus, as well as the resulting social disruption and physical distancing due to the pandemic, have had the same effect [27,28,29].

The previous points justify the feelings of uncertainty during the COVID-19 pandemic [30].

In Greece, the first COVID-19 case was diagnosed on 26 February 2020, and the first death was reported on 12 March 2020. As of 29 April 2021, 337,723 confirmed cases and 10,179 deaths have been reported in Greece [31]. A few weeks after the first cases of COVID-19, strict containment measures were introduced, including the closure of schools, universities, non-essential shops, cafes and restaurants, public spaces, as well as movement restrictions including a ban on gatherings and travel [32].

Because uncertainty is considered one of the more obvious consequences of lockdowns [33], based on the previous points, the objective of this study was to identify the individual characteristics associated with a feeling of uncertainty during the lockdown that commenced in March 2020 in Greece.

## 2. Materials and Methods

For the purpose of this study, data from the “Public Opinion in the EU in Time of Coronavirus Crisis” [34] survey were used. The survey was conducted using Kantar’s online access panel between 23 April and 1 May 2020, among 21,804 respondents in 21 EU Member States. The survey was limited to respondents aged between 16 and 64 years. In some countries, including Greece, the sample was limited to respondents aged between 16 and 54 years. Representativeness at the national level was ensured by quotas concerning gender, age, and region. The sample size was *n* = 1050 in Greece. The data collection took place between 23 April and 27 April 2020.

The business and workplace suspensions implemented in Greece between 10 March and 18 March 2020 included the following: (a) closure of schools and universities on 10 March; (b) closure of movie theaters, courtrooms, and gyms on 12 March; (c) closure of malls, cafés, restaurants, bars, museums, archaeological sites, and beauty parlors on 13 March; (d) closure of all organized beaches and ski resorts on 14 March; (e) closure of all stores with the exception of supermarkets and pharmacies on 18 March. In addition, a nationwide restriction of movement was imposed on 23 March (3 May was the last day of the lockdown). Business restrictions were gradually relaxed up to the end of May [35].

The variable under study was whether or not uncertainty is among the feelings that best describe respondents’ current emotional status. The respondents were asked the question “What feelings best describe your current emotional status?” One of the response options was “uncertainty”, and the remaining response options were (a) frustration, (b) hope, (c) fear, (d) anger, (e) helpfulness, (f) confidence, (g) helplessness, and (h) other. Respondents could choose a maximum of three different response options. From the abovementioned variable, a dichotomous variable, i.e., the response, was derived as follows: (a) uncertainty was among the feelings that best described respondents’ current emotional status (1), i.e., in this case, respondents chose uncertainty either alone or in combination with other feelings (one or two); and (b) uncertainty was not among the feelings that best described respondents’ current emotional status (0), i.e., in this case, respondents chose exclusively other feelings.

Thus, as in Powell et al.’s article (2007) [36], in this study, uncertainty is defined as an individual’s perception. Because a person who believes himself or herself to be uncertain is uncertain [3], the responses in this study reflect the self-perceived event of uncertainty.

Because the response was binary, a logistic regression model was used. The potential predictors used in the analysis are presented in Table 1.

Social class was based on the occupation of the main earner of the household: (a) 1: low–semi-skilled or unskilled manual workers, students, retired and living on state pension only, unemployed (for over six months), or not working due to long-term sickness; (b) 2: middle–skilled manual workers, supervisory or clerical/junior managerial/professional/administrator; (c) 3: high–intermediate managerial/professional/administrative, higher managerial/professional/administrative.

Helmert coding was applied to the ordinal variables (a) social class, (b) COVID-19-related concern for one’s own health, and (c) COVID-19-related concern for one’s family and friends’ health. The Helmert contrast compares each category of an ordinal variable (except the last) with the mean of the subsequent levels. Indicator coding was applied to the following nominal variables: (a) region, (b) marital status, and (c) current employment status. Indicator contrast was used to compare the reference category of a nominal variable with the remaining categories. The binary variables “experiencing loss of income since the beginning to the COVID-19 pandemic”, “experiencing unemployment or partial unemployment since the beginning of the COVID-19 pandemic”, “gender”, and “presence of children” were treated as such.

The model’s goodness of fit was tested using the Hosmer and Lemeshow test. The calibration of the model was tested using the calibration belt test. In addition, the model was tested for specification error using the link test.

The STATA 14 statistical software package was used for the analysis. Specifically, the commands desmat [37], logistic, linktest, and calibrationbelt [38] were used.

## 3. Results

Regarding gender, 50% of the respondents were female, and 50% were male. The mean age of the respondents was 37.14 years (±10.47), while 17.05% of the respondents were aged between 16 and 24 years, 22.29% between 25 and 34 years, 29.90% between 35 and 44 years, and 30.76% between 45 and 54 years. The respondents’ characteristics are presented in Table 2.

According to the descriptive analysis, 69.13% of the respondents of the survey declared that uncertainty was among the feelings that best described their current emotional status. Uncertainty was ranked first among the nine feelings.

In addition, 41.33% of the respondents had experienced loss of income since the beginning of the COVID-19 pandemic, and 28.29% of the respondents had experienced unemployment or partial unemployment.

Furthermore, it is worth noting that only 11.35% of the respondents were very concerned for their own health, and 23.29% of the respondents were very concerned for the health of their family and friends (Table 3).

According to the results shown in Table 4, uncertainty was among the feelings that best described the current emotional status of older individuals (OR = 1.015 (>1), 95% Confidence Interval (CI): 1.001–1.030), as well as of those experiencing income loss (OR = 1.386 (>1), 95% CI: 1.031–1.865), those experiencing unemployment or partial unemployment (OR = 1.903 (>1), 95% CI: 1.348–2.686), those who were not very concerned for their own health as compared with those who were not at all concerned for their own health (OR = 1.621 (>1), 95% CI: 1.033–2.544), those who were not very concerned for family and friends’ health as compared with those who were not at all concerned for family and friends’ health (OR = 2.030 (>1), 95% CI: 1.124–3.667), those who were fairly concerned for family and friends’ health as compared with those who were not very concerned for family and friends’ health and those who were not at all concerned for family and friends’ health (OR = 2.257 (>1), 95% CI: 1.495–3.407), and those who were very concerned for family and friends’ health as compared with those who were fairly concerned for family and friends’ health, those who were not very concerned for family and friends’ health, and those who were not at all concerned for family and friends’ health (OR = 2.725 (>1), 95% CI: 1.713–4.333). However, it is less likely that uncertainty was among the feelings that best described the current emotional status of lower-social-class individuals (OR = 0.604 (<1), 95% CI: 0.437–0.833) as compared with middle- and higher-social-class individuals.

According to the link test (Table 5), the model did not suffer from specification error.

In addition, both the Hosmer and Lemeshow tests (*p* = 0.102) and the calibration belt test (*p* = 0.375) indicated a good fit.

Based on the logistic regression model, income and job losses since the beginning of the pandemic were linked to increased odds of feeling uncertainty during the lockdown.

## 4. Conclusions

The COVID-19 pandemic has resulted in significant uncertainty for the global population [39].

According to the results of this study, the probability that uncertainty is among the feelings that best describe individuals’ emotional status depends on age, income and job losses, social class, concern for own health and concern for family and friends’ health. The influence of both economic factors (income and job losses) and health-related factors (concern for health) indicates that uncertainty arises from various aspects of the COVID-19 crisis, i.e., economic impacts, health impacts, and social impacts.

In regard to age, the explanation for the positive association (OR = 1.015 > 1) with feelings of uncertainty may be that for this particular COVID-19 viral infection, age increases vulnerability, even prior to the age of 65 [40]. The epidemiologic data and daily information in Greece are consistent with this evidence. As of 27 April 2020, the mean age of COVID-19 confirmed cases was 49 years, and the mean age of COVID-19-related deaths was 74 years [41].

Furthermore, there is a positive association between income loss and the probability that uncertainty is one of the feelings that best describes individuals’ current emotional status, since OR = 1.386 (>1). Similarly, job loss was positively associated with the probability that uncertainty is among the feelings that best describe individuals’ current emotional status, since OR = 1.903 (>1). The plausible explanation is that during the COVID-19 pandemic, individuals have been exposed to increasing job and financial insecurities [42,43] due to job and income losses [44], which have been associated with the deterioration of security and stability of individuals’ personal finances, all of which could be linked to strong feelings of uncertainty [45].

Because income and job losses are directly associated with financial uncertainty, as mentioned above, the results of this study are consistent with the literature. Furthermore, in combination with the unpredictability of illness, which may expose individuals to unexpected financial expenses [46], the potential but realistic threat of future income and job losses generate additional financial uncertainty.

In addition, because health emergencies lead to economic impacts through (a) unanticipated healthcare costs, (b) forced limitation of other essential expenditure to meet healthcare costs, and (c) income losses through inability to work, individual illness, or caring for another person [47], it is not surprising that income and job losses are positively associated with the probability of feeling uncertain.

The literature indicates that an individual’s vulnerability to uncertainty depends largely on that individual’s place in society [48]. As such, one would expect that uncertainty would be among the feelings that best describe the emotional status of lower-class individuals. According to the results, this was not confirmed, since the OR of lower-social-class individuals as compared with middle- and higher-social-class individuals was lower than one (OR = 0.604). Thus, although limited resources and uncertainty characterize the social context of lower-class individuals in general [49], it seems that in the case of COVID-19, the feelings of uncertainty that restrictions and lockdowns generate [50] may have another predictor. One explanation may be health literacy.

Health literacy is defined as “the degree to which individuals have the capacity to obtain, process, and understand basic health information and services needed to make appropriate health decisions” [51]. It is evident that limited health literacy exists among those whose social status is very low or low [52].

Due to the high degree of multidimensional uncertainty, interpreting COVID-19-related news and official recommendations is of particular difficulty. Integrating this large volume of information into behavioral actions is a task that requires critical health literacy and becomes a significant challenge for individuals [53]. In other words, in this uncertain era, health literacy is a key factor for survival [54]. Because it is evident that the health literacy of those who believe they are less likely to be infected with COVID-19 is lower [55], we may argue that individuals with limited health literacy, including lower-class individuals, may have a limited capacity to conceive the uncertainties surrounding the COVID-19 pandemic.

Finally, the emotional status of individuals with higher levels of concern for their own health and higher levels of concern for family and friends’ health is more likely to be described as a feeling of uncertainty. Specifically, in regard to the concern for one’s own health, it is more likely that uncertainty is one of the feelings that best describe the current emotional status of those who are not very concerned for their own health as compared with those who are not at all concerned for their own health, since OR was equal to 1.621 (>1). This same trend of a higher likelihood of feeling uncertain is evident for the concern that individuals have about their family and friend’s health (OR = 2.030 > 1, OR = 2.257 > 1, OR = 2.725 > 1).

Because it is evident that one’s fears and concerns affect his/her emotions, i.e., he/she may feel uncertain, depressed, anxious, etc. [56], the results regarding the influence of concern for health agree with the international literature. That is, feelings of uncertainty are also generated by the unpredictability of who will get infected or become sick, the duration of the pandemic, and its short-term or long-term effects [57]. Therefore, the pandemic period represents a long-lasting highly stressful event involving constant and prolonged feelings of uncertainty and worry related to the risk of infection [58].

The aim of this study was to confirm the complexity of the feelings of uncertainty experienced by individuals during a lockdown and that such feelings result from the direct effects of the pandemic, i.e., changes in individuals’ personal finances and concerns for one’s own health and for that of family and friends. Since uncertainty arises from various aspects of the COVID-19 pandemic, namely, economic, health-related, and social, this study integrates the way that several sources of uncertainty related to the COVID-19 pandemic translate to feelings of uncertainty.

The uncertainty generated by the COVID-19 pandemic over the short and long term is likely to have different effects on different sociodemographic groups. Furthermore, these effects are also likely to be modified by a country’s welfare system and the emergency interventions of its institutions [59]. Although uncertainty cannot be eliminated, better management would help to minimize its damage [60].

The previous discussion indicates that it is clear that policy is vital for coping with the COVID-19 pandemic. However, the formation of COVID-19 policy must address the uncertainties surrounding the nature of the disease, the dynamics of transmission, and behavioral responses. That is, to make useful predictions of policy impacts and reasonable policy decisions, a credible means of measuring COVID-19 uncertainty is needed [61]. Because policies should compromise between preventing an outbreak that would overwhelm domestic resources and mitigating economic loss [62], this evaluation is a difficult task.

## Figures and Tables

**Table 1:** Potential predictors.

Variable	Category	Code (Range)
Geographic Region	Attica	1
	Macedonia and Thrace	2
	Epirus and Western Macedonia	3
	Thessaly and Central Greece	4
	Peloponnese, Western Greece and Ionian Islands	5
	Aegean Islands and Crete	6
Gender	Female	0
	Male	1
Age		16–54
Marital Status	Married/Living with Partner	1
	Never Married (Single)	2
	Divorced/Widowed	3
	Living with Parents	4
	Domestic Partner/Living with Other Adults	5
Presence of Children	No	0
	Yes	1
Social Class	Low	1
	Middle	2
	High	3
Experiencing Loss of Income since the Beginning of the COVID-19 Pandemic	No	0
	Yes	1
Experiencing Unemployment or Partial Unemployment since the Beginning of the COVID-19 Pandemic	No	0
	Yes	1
Current Employment Status	Employed (Full-Time)	1
	Employed (Part-Time)	2
	Self-Employed	3
	Retired/Unable to Work/Disabled	4
	Still at School	5
	In Full-Time Higher Education	6
	Unemployed and Seeking Work	7
	Not Working and Not Seeking Work	8
COVID-19-Related Concern for Own Health	Very Concerned	1
	Fairly Concerned	2
	Not Very Concerned	3
	Not at All Concerned	4
COVID-19-Related Concern for Family and Friends’ Health	Very Concerned	1
	Fairly Concerned	2
	Not Very Concerned	3
	Not at All Concerned	4

**Table 2:** Respondents’ Characteristics.

Age	Gender % (*n*)	
	Male	Female
16–24	8.57 (90)	8.48 (89)
25–34	11.05 (116)	11.24 (118)
35–44	14.95 (157)	14.95 (157)
45–54	15.43 (162)	15.33 (161)

**Table 3:** Concern for Health.

Category	Concern for Own Health % (*n*)	Concern for Family and Friends’ Healtth % (*n*)
Very Concerned	11.35 (118)	23.29 (241)
Fairly Concerned	35.29 (367)	47.44 (491)
Not Very Concerned	37.98 (395)	21.74 (225)
Not at All Concerned	15.38 (160)	7.54 (78)

**Table 4:** Logistic regression model.

Variable	OR	*p*	95% CI	
Age	1.015	0.030	1.001	1.030
Income Loss	1.386	0.031	1.031	1.865
Job Loss	1.903	<0.001	1.348	2.686
Social Class		0.007		
Low vs. Middle and High	0.604	0.002	0.437	0.833
Middle vs. High	1.059	0.739	0.757	1.481
COVID-19-Related Concern for own Health		0.038		
Very Concerned vs. Subsequent Levels	0.637	0.097	0.374	1.084
Fairly Concerned vs. Subsequent Levels	1.106	0.603	0.757	1.616
Not Very Concerned vs. Not at all Concerned	1.621	0.036	1.033	2.544
COVID-19-Related Concern for Health of Family and Friends		<0.001		
Very Concerned vs. Subsequent Levels	2.725	<0.001	1.713	4.333
Fairly Concerned vs. Subsequent Levels	2.257	<0.001	1.495	3.407
Not Very Concerned vs. Not at all Concerned	2.030	0.019	1.124	3.667
Constant	0.695	0.198	0.400	1.210

**Table 5:** Link Test.

Variable	Coefficient	*p*	95% Confidence Interval	
h	0.879	<0.001	0.540	1.218
h^2^	0.104	0.376	−0.127	0.336
Constant	−0.012	0.911	−0.230	0.205

## Data Availability

The data are available by the Public Opinion Monitoring Unit. Directorate- General for Communication. European Parliament.
